# Randomized phase I clinical trial of anti–α‐synuclein antibody BIIB054

**DOI:** 10.1002/mds.27738

**Published:** 2019-06-17

**Authors:** Miroslaw Brys, Laura Fanning, Serena Hung, Aaron Ellenbogen, Natalia Penner, Minhua Yang, Mackenzie Welch, Erica Koenig, Eric David, Tara Fox, Shavy Makh, Jason Aldred, Ira Goodman, Blake Pepinsky, YuTing Liu, Danielle Graham, Andreas Weihofen, Jesse M. Cedarbaum

**Affiliations:** ^1^ Biogen Cambridge Massachusetts USA; ^2^ Michigan Institute for Neurological Disorders Farmington Hills Michigan USA; ^3^ QUEST Research Institute Farmington Michigan USA; ^4^ Biogen Maidenhead UK; ^5^ Selkirk Neurology & Inland Northwest Neurological Spokane Washington USA; ^6^ The Compass Clinic Orlando Florida USA

**Keywords:** Parkinson's disease, pharmacokinetics, phase I, synucleinopathy, α‐synuclein

## Abstract

**Background:**

Pathological and genetic evidence implicates toxic effects of aggregated α‐synuclein in the pathophysiology of neuronal dysfunction and degeneration in Parkinson's disease. Immunotherapy targeting aggregated α‐synuclein is a promising strategy for delaying disease progression.

**Objective:**

This study (NCT02459886) evaluated the safety, tolerability, and pharmacokinetics of BIIB054, a human‐derived monoclonal antibody that preferentially binds to aggregated α‐synuclein, in healthy volunteers and participants with Parkinson's disease.

**Methods:**

A total of 48 healthy volunteers (age 40–65, 19 women) and 18 Parkinson's disease participants (age 47–75, 5 women, Hoehn and Yahr stage ≤2.5) were in the study. Volunteers were enrolled into 6 single‐dose cohorts of BIIB054 (range 1–135 mg/kg) or placebo, administered intravenously; Parkinson's disease participants received a single dose of BIIB054 (15 or 45 mg/kg) or placebo. All participants were evaluated for 16 weeks with clinical, neuroimaging, electrocardiogram, and laboratory assessments. Serum and cerebrospinal fluid BIIB054 concentrations were measured. BIIB054/α‐synuclein complexes were measured in plasma.

**Results:**

Most adverse events were mild and assessed by investigators as unrelated to the study drug. Pharmacokinetic parameters for volunteers and the Parkinson's disease participants were similar. BIIB054 serum exposure and maximum concentrations were dose proportional during the dose range studied. In volunteers and the Parkinson's disease participants, the serum half‐life of BIIB054 was 28 to 35 days; the cerebrospinal fluid–to‐serum ratio ranged from 0.13% to 0.56%. The presence of BIIB054/α‐synuclein complexes in plasma was confirmed; all Parkinson's disease participants showed almost complete saturation of the BIIB054/α‐synuclein complex formation.

**Conclusions:**

BIIB054 has favorable safety, tolerability, and pharmacokinetic profiles in volunteers and Parkinson's disease participants, supporting further clinical development. © 2019 The Authors. *Movement Disorders* published by Wiley Periodicals, Inc. on behalf of International Parkinson and Movement Disorder Society.

Parkinson's disease (PD) is the second most common neurodegenerative disorder.^1^ It is estimated that the number of people with PD will double from 6.9 million in 2015 to 14.2 million in 2040.^2^ Current treatments provide only partial relief of motor symptoms and may worsen other symptoms.^3^ There are no approved therapies that slow disease progression or adequately treat levodopa‐unresponsive symptoms (eg, cognitive or autonomic).

Histopathological hallmarks of PD include presence of Lewy bodies and Lewy neurites that are primarily composed of aggregated α‐synuclein (α‐syn).^4^ Furthermore, genetic studies point to a causal role of α‐syn in PD, as mutations and duplications of the gene coding for α‐syn, *SNCA,* have been linked to inherited forms of PD. These observations suggest that α‐syn is a promising therapeutic target in PD, and increasing numbers of new therapies targeting α‐syn are in development.^5^

Monoclonal antibodies that target α‐syn have been shown to reduce α‐syn pathology and ameliorate behavioral deficits in animal models.^6^, ^7^, ^8^ BIIB054 is a human‐derived monoclonal antibody targeting α‐syn that was generated from a library of memory B cells from elderly individuals with no signs of neurodegenerative disorders.^9^ The antibody was engineered with human glycosylated immunoglobulin G1 heavy‐chain and lambda light‐chain constant region sequences and produced in Chinese hamster ovary cells. BIIB054 has a ≥800‐fold greater apparent affinity for pathologic aggregated α‐syn than for the more abundant physiological monomeric protein.^9^ In animal models, BIIB054 treatment attenuated the spreading of α‐syn pathology, rescued motor impairments, and reduced the loss of dopamine transporter density in dopaminergic terminals in striatum,^9^ suggesting that BIIB054 has the potential to mitigate α‐syn‐mediated pathological changes and thereby slow the progression of PD. Here, we report the results of the first‐in‐human study of BIIB054.

## Methods

### Objectives

The primary objective of the study was to assess the safety and tolerability of single doses of BIIB054 in healthy volunteers (HVs) and participants with early PD. Secondary objectives included the assessment of the pharmacokinetics and immunogenicity of BIIB054. We also explored the engagement of α‐syn with BIIB054.

### Study Material: Antibody

BIIB054 was produced at Biogen (Durham, North Carolina) under current good manufacturing practice procedures and supplied to clinical sites for intravenous administration (data on file, Biogen).

### Study Design and Participants

This was a 2‐part, phase I, randomized, double‐blind, placebo‐controlled, single‐ascending dose study (NCT02459886). Ethics committee approval of the protocol was obtained by investigators, and the study was performed in accordance with good clinical practice and the Declaration of Helsinki. All participants provided written informed consent. Part 1 was conducted July 1, 2015 through November 30, 2016 at 2 U.S. sites and enrolled 48 HVs aged 40 to 65 years with a body mass index of 19 to 30 kg/m^2^ (inclusive) and no history of cardiovascular disease or significant abnormalities on electrocardiogram. Participants were excluded if they tested positive for drugs or alcohol at screening; smoked >5 cigarettes daily; used prescription or over‐the‐counter products (excluding acetaminophen, hormone replacement therapy, birth control); had clinically significant abnormal laboratory test values including alanine and aspartate aminotransferases, bilirubin, or creatinine above the upper limit of normal; or low hemoglobin (<12 g/dL, men; <11 g/dL, women) or platelet levels. Part 2 was conducted February 7 through November 20, 2017 at 7 U.S. sites and enrolled 18 participants aged 40 to 80 years with idiopathic PD, a body mass index of 19 to 32 kg/m^2^ (inclusive), Hoehn and Yahr stage ≤2.5, and time since PD diagnosis ≤5 years. PD diagnosis was based on the presence of bradykinesia plus either resting tremor or rigidity according to the United Kingdom Parkinson's Disease Society Brain Bank clinical diagnostic criteria.^10^ In addition, the participants could not have motor fluctuations or dyskinesias. The participants were either treatment naive and not expected to initiate symptomatic therapy during the study or could be on a stable low dose of symptomatic medications for ≥8 weeks (including selegiline ≤5 mg twice daily, rasagiline ≤1 mg once daily, or immediate‐release carbidopa/levodopa ≤25/100 mg 3 times daily).

Additional exclusion criteria included other active medical conditions, the use of any typical or atypical antipsychotics or metoclopramide, the presence of freezing of gait, clinically significant structural brain disease on magnetic resonance imaging (MRI), significant cognitive impairment, and history of transient ischemic attack, stroke, or unexplained loss of consciousness within the past year. Full inclusion and exclusion criteria are listed in the Supplementary Materials.

**Figure 1:**
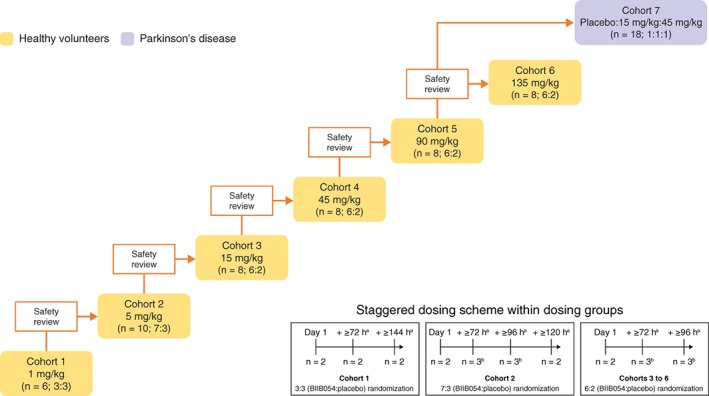
Study design. Staggered dosing was not used in cohort 7. ^a^Time from completion of dosing of first participant within same cohort. ^b^Up to 3 participants.

In part 1, HVs were assigned to 6 sequential dose cohorts to receive single intravenous infusions of BIIB054 or saline placebo (Fig. 1). The BIIB054 cohorts were as follows: 1 mg/kg (n = 6), 5 mg/kg (n = 10), and 15, 45, 90, and 135 mg/kg (n = 8/cohort) and randomly assigned in 3:3, 7:3, and 6:2 ratios, respectively. Randomization, generated by an independent statistician, was staggered within each dose group: for the 1‐mg/kg group, the first 2 participants were randomly assigned 1:1 via block randomization to a single dose of BIIB054 or placebo. After 72 hours of blinded safety data were reviewed, 2 more participants were randomly assigned. After 72 hours of safety data on the additional 2 participants were reviewed, the last 2 participants were randomly assigned. Randomization was similarly staggered for the other dose groups. Blinded safety data for ≥28 days from each dose group were reviewed before the assignment of participants to the next dose group. Part 2 participants (with PD) were randomly assigned (1:1:1) in parallel to receive a single intravenous dose of BIIB054 15 or 45 mg/kg or placebo. Study drug/infusion solution for both parts was prepared by unblinded pharmacists and administered via intravenous infusion (duration up to 2 hours) by blinded staff. Except for a limited sponsor's safety surveillance team (including medical director, safety and benefit risk physician, biostatistician, and clinical pharmacologist), all other staff were blinded to treatment assignments. Two significant design changes occurred after study start: the addition of the 135‐mg/kg HV cohort (February 17, 2016) and the early PD cohort (April 13, 2016).

### Procedures

Participants were admitted to the clinical site 2 days before study drug administration and remained there for approximately 72 hours after dosing. Serum samples for pharmacokinetic analysis were collected before study drug administration, throughout 72 hours after administration, and during weekly visits through week 16 and for assessment of anti‐BIIB054 antibodies at weeks 4, 6, 12, and 16. All participants underwent a lumbar puncture 1 to 2 days before study drug administration to collect cerebrospinal fluid (CSF). HVs underwent additional lumbar punctures 8 hours postadministration, on day 2, and at week 3. PD participants underwent lumbar punctures at weeks 1 and 4. Brain MRIs for all participants were acquired during the 28‐day screening period, on day 3, and at week 4. MRIs included, at a minimum: T1, fluid‐attenuated inversion recovery and diffusion‐weighted imaging. Participants were monitored for adverse events (AEs), clinical laboratory tests, vital sign measurements, and electrocardiogram measurements. The Columbia Suicide Severity Rating Scale, Montreal Cognitive Assessment, and neurological and physical examinations were administered at specified intervals throughout the study. PD participants underwent dopamine transporter single‐photon emission computed tomography scan (DaTscan) during screening and were assessed on the full Movement Disorders Society–Unified Parkinson's Disease Rating Scale (MDS‐UPDRS)^11^ at baseline and end of study and with part III (motor) on day 4 and at weeks 4 and 12. The Nonmotor Symptoms Scale^12^ and Scale for Outcomes in Parkinson's Disease for Autonomic Symptoms^13^ were assessed in PD participants 1 to 2 days before study drug administration, on day 4, and at weeks 4, 12, and 16.

### Outcomes

The safety and tolerability of BIIB054 were assessed by AE incidence, physical and neurologic examinations, and clinical laboratory tests. AEs were coded to system organ class and preferred term using the Medical Dictionary for Regulatory Activities, version 20.0. Serum pharmacokinetic endpoints for BIIB054 were computed using Phoenix WinNonlin 6.4 software (Certara, Princeton, New Jersey) and included area under the concentration‐time curve from 0 extrapolated to infinity (AUC_0‐inf_), AUC from 0 to last measurable concentration (AUC_0‐tlast_), maximum concentration (C_max_), time to C_max_, elimination half‐life (t_½_), clearance (CL), and volume of distribution at steady state. For the assessment of immunogenicity, a bridging immunoassay was used to detect anti‐BIIB054 antibodies. Samples were tested in a tiered fashion. All samples were initially evaluated in a screening tier; samples that were positive (signal equal to or above a statistically determined cutpoint) were further evaluated in a confirm tier to determine the specificity of the response. Samples that confirm positive (signal equal to or above a statistically determined cutpoint) were evaluated in the titration assay to determine magnitude of the immune response. Engagement of BIIB054 with α‐syn in peripheral blood was assessed by measurement of total α‐syn (ie, α‐syn bound and not bound to BIIB054) and by quantification of BIIB054/α‐syn complex concentrations in plasma. BIIB054 and total α‐syn concentrations in serum and CSF were measured using validated enzyme‐linked immunosorbent assays. BIIB054/α‐syn complex concentrations in serum were measured using a quantitative size exclusion chromatography method (details in the Supplementary Materials).

### Statistical Analysis

The sample size for this study was not determined based on statistical considerations but, rather, based on typical cohort size in similar phase I studies. With a total of 34, 12, and 46 participants dosed with BIIB054 (HV, PD, and HV and PD combined, respectively), there was a ≥80% probability of observing at least 1 AE for AEs with incidence rates of 4.6%, 12.6%, and 3.4%, or higher, respectively. HV and PD participants were analyzed separately. Safety was assessed in all participants who were randomly assigned and received study treatment. Pharmacokinetics, immunogenicity, and pharmacodynamics were assessed in the respective populations, which consisted of all participants who were randomly assigned, received study treatment, and had at least 1 pharmacokinetic, immunogenicity, or pharmacodynamic measurement, respectively, after baseline. Given the small sample size, descriptive statistics were used to summarize the safety data; continuous data were summarized by treatment group and/or timepoint (if relevant) using descriptive statistics including mean and standard deviation or percent coefficient of variation. Categorical data were summarized by treatment group and/or timepoint (if relevant) using frequencies and percentages. Change from baseline in log‐transformed α‐syn concentration over time was analyzed with a mixed‐effect repeated measures model with treatment group, visit, and treatment‐group‐by‐visit interaction terms and adjusted for baseline values. α‐syn AUC was analyzed using an analysis of variance adjusting for baseline concentration. All statistical analyses were performed using SAS statistical software, version 9.4 (SAS Institute, Cary, North Carolina).

## Results

### Participants

**Figure 2:**
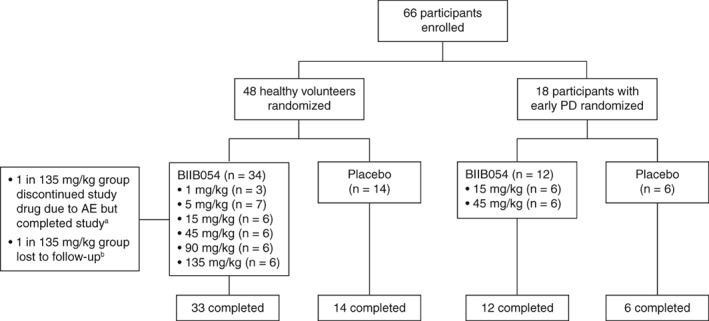
Patient disposition. ^a^Participant received 32% of BIIB054 dose because of grade 1 hypersensitivity reaction but completed study. ^b^Participant could not be contacted after the week 3 visit despite multiple phone calls and certified letters, was listed as lost to follow‐up, and did not complete the study. AE, adverse event; PD, Parkinson's disease.

A total of 47 of 48 HVs and all of the PD participants completed the study; 1 HV in the BIIB054 135‐mg/kg group could not be contacted after week 3 and was considered lost to follow‐up (Fig. 2). The demographics in HVs and PD participants were comparable across dose groups, although PD participants were slightly older than HVs (Table 1): median (range) age was 50 (40–65) and 64 (47–75) years in the HVs and PD participants, respectively. Male PD participants outnumbered the females, consistent with PD demographics. Among the HVs, 75% were white, whereas all PD participants were white. Baseline MDS‐UPDRS (Table 1), Nonmotor Symptoms Scale, and Scale for Outcomes in Parkinson's Disease for Autonomic Symptoms scores (data not shown) were comparable across treatment groups in the PD participants. All of the PD participants had Hoehn and Yahr scores of 1 or 2. Of the PD participants, 4 (67%), 3 (50%), and 2 (33%) took concomitant PD medications in the placebo, 15‐mg/kg, and 45‐mg/kg groups, respectively (Table 1). With the exception of 1 PD participant in the placebo group, all other PD participants had evidence of dopaminergic deficit on DaTscan. The participant with a negative DaTscan scan was not excluded from the analysis. Because safety, tolerability, and pharmacokinetics were the primary goals of the study, the protocol allowed up to 2 participants with negative DaTscans to enter the study (see the Supplementary Materials for study inclusion criteria).

**Table 1:** Baseline characteristics

Characteristic	Healthy volunteers							Participants with PD		
	Placebo, n = 14	BIIB054						Placebo, n = 6	BIIB054	
		1 mg/kg, n = 3	5 mg/kg, n = 7	15 mg/kg, n = 6	45 mg/kg, n = 6	90 mg/kg, n = 6	135 mg/kg, n = 6		15 mg/kg, n = 6	45 mg/kg, n = 6
Age, y, mean (range)	53.5 (40–65)	51.0 (46–59)	50.6 (43–61)	45.0 (41–52)	53.0 (48–63)	48.2 (40–60)	51.2 (43–59)	66.2 (51–75)	63.7 (51–72)	57.7 (47–69)
Male, n (%)	8 (57)	1 (33)	3 (43)	4 (67)	4 (67)	4 (67)	5 (83)	4 (67)	4 (67)	5 (83)
Race, n (%)										
White	10 (71)	1 (33)	4 (57)	5 (83)	6 (100)	4 (67)	6 (100)	6 (100)	6 (100)	6 (100)
Black/African American	4 (29)	2 (67)	3 (43)	1 (17)	0	2 (33)	0	0	0	0
Weight, kg, mean	79.74	79.97	74.37	78.32	78.88	78.23	70.43	82.42	84.75	80.53
BMI, kg/m^2^, mean (range)	26.57 (22.7–30.2)	26.92 (25.7–27.6)	25.67 (20.5–30.9)	26.57 (22.6–28.8)	27.17 (26.2–28.3)	27.06 (22.9–29.7)	24.44 (21.5–29.6)	26.75 (23.8–30.0)	27.39 (24.4–29.5)	25.83 (21.2–31.8)
H&Y score, n (%)										
1	NA	NA	NA	NA	NA	NA	NA	0 (0)	1 (17)	2 (33)
2	NA	NA	NA	NA	NA	NA	NA	6 (100)	5 (83)	4 (67)
MDS‐UPDRS, mean/median (range)										
I	NA	NA	NA	NA	NA	NA	NA	4.7/2.5 (1–14)	5.7/5.0 (2–12)	6.3/5.5 (0–16)
II	NA	NA	NA	NA	NA	NA	NA	7.8/7.5 (2–14)	3.8/4.0 (1–6)	6.7/6.0 (1–17)
IIII	NA	NA	NA	NA	NA	NA	NA	27.5/29.5 (12–39)	24.2/24.0 (14–33)	20.2/19.0 (9–37)
IV	NA	NA	NA	NA	NA	NA	NA	1.3/0.0 (0–6)	0.5/0.0 (0–3)	0.5/0.0 (0–3)
Symptomatic treatment, n (%)	NA	NA	NA	NA	NA	NA	NA	4 (67)	3 (50)	2 (33)
Carbidopa/levodopa								2	2	0
Rasagiline								1	1	1
Both								1	0	1

BMI, body mass index; H&Y, Hoehn and Yahr; MDS‐UPDRS, Movement Disorders Society–Unified Parkinson's Disease Rating Scale; NA, not available; PD, Parkinson's disease.

### Safety

#### Part 1 (HV)

Of the HVs, 19 of 34 (56%) administered BIIB054 and 7 of 14 (50%) administered placebo experienced at least 1 treatment‐emergent AE (Table 2). The overall incidence of AEs was similar in those administered BIIB054 at ≤45 mg/kg and higher in those who received 90 and 135 mg/kg versus those administered the placebo. The percentages of HVs experiencing particular AEs were comparable across all treatment groups. The most common AEs were headache, dizziness, and procedural pain. A total of 6 HVs experienced AEs considered by the investigator to be treatment‐related, including dysgeusia (n = 1, placebo), diarrhea (n = 1, placebo), asymptomatic ventricular tachycardia (n = 1, 15 mg/kg), headache (n = 1, 90 mg/kg), and hypersensitivity reaction (n = 1, 135 mg/kg). Only 1 serious AE, assessed by the investigator as treatment related, was reported: a HV (51‐year‐old male with history of smoking, palpitations, and no family history of vascular disease) in the 135‐mg/kg group developed a 5‐mm area of restricted diffusion in his right parietal lobe on the 4‐week postdose routine MRI consistent with an asymptomatic cerebrovascular accident that was not present on his predose scan. The participant did not report any symptoms, and his neurological examination was normal and remained normal throughout the study. This AE was considered related to study treatment by the investigator.

**Table 2:** Adverse events

AE, n (%)	Healthy volunteers								Participants with PD			
	Placebo, n = 14	BIIB054							Placebo, n = 6	BIIB054		
		All, n = 34	1 mg/kg, n = 3	5 mg/kg, n = 7	15 mg/kg, n = 6	45 mg/kg, n = 6	90 mg/kg, n = 6	135 mg/kg, n = 6		All, n = 12	15 mg/kg, n = 6	45 mg/kg, n = 6
Any AE	7 (50)	19 (56)	1 (33)	4 (57)	2 (33)	2 (33)	5 (83)	5 (83)	6 (100)	9 (75)	5 (83)	4 (67)
AEs in ≥10% in placebo or all BIIB054												
Headache	4 (29)	8 (24)	1 (33)	2 (29)	0	1 (17)	3 (50)	1 (17)	2 (33)	4 (33)	2 (33)	2 (33)
Dizziness	2 (14)	5 (15)	1 (33)	1 (14)	0	1 (17)	1 (17)	1 (17)	0	0	0	0
Procedural pain	1 (7)	4 (12)	0	1 (14)	0	1 (17)	1 (17)	1 (17)	0	0	0	0
Back pain	1 (7)	2 (6)	0	0	0	0	1 (17)	0	0	2 (17)	1 (17)	1 (17)
Post–LP syndrome	0	0	0	0	0	0	0	0	0	2 (17)	1 (17)	1 (17)
Upper respiratory tract infection	0	0	0	0	0	0	0	0	0	2 (17)	1 (17)	1 (17)
CTCAE grade												
2	1 (7)^a^	5 (15)^b^	1 (33)^b^	0	1 (17)	0	3 (50)^b^	0	2 (33)^c^	4 (33)^d^	2 (33)^d^	2 (33)^d^
3	0	1 (3)^e^	0	0	0	0	0	1 (17)^e^	1 (17)^f^	0	0	0
AEs related to study treatment	2 (14)	4 (12)^g^	0	0	1 (17)	0	1 (17)	2 (33)^g^	1 (17)^f^	1 (8)	0	1 (17)
Serious AEs	0	1 (3)^e^	0	0	0	0	0	1 (17)^e^	1 (17)^f^	0	0	0

AE, adverse event; CTCAE, Common Terminology Criteria for Adverse Events; LP, lumbar puncture; PD, Parkinson's disease.

a Foot fracture and pain.

b One healthy volunteer who received BIIB054 1 mg/kg and 2 who received BIIB054 90 mg/kg experienced grade 2 headaches; 1 healthy volunteer who received BIIB054 15 mg/kg experienced a grade 2 episode of ventricular tachycardia while sleeping; and 1 healthy volunteer who received BIIB054 90 mg/kg experienced grade 2 dyspepsia.

c One participant experienced a grade 2 headache, and 1 participant experienced a grade 2 ear infection.

d One participant who received BIIB054 15 mg/kg had a grade 2 post–LP syndrome, and 1 participant who received BIIB054 15 mg/kg experienced a grade 2 headache and grade 2 constipation; 1 participant who received 45 mg/kg BIIB054 experienced a grade 2 bone contusion; and 1 participant who received BIIB054 45 mg/kg experienced 2 grade 2 headaches and grade 2 back pain.

e Asymptomatic cerebrovascular accident considered related to study treatment.

f Asthma exacerbation considered unrelated to study treatment.

g One healthy volunteer who received BIIB054 135 mg/kg experienced a hypersensitivity reaction considered related to the study drug and discontinued study drug after administration of 163 mL (32%) of the 508‐mL infusion.

#### Part 2 (Participants With Early PD)

AEs were reported in 9 (75%) PD participants who received BIIB054 and 6 (100%) who received placebo. A total of 2 participants (1 administered placebo; 1 administered 45 mg/kg) experienced headache, which was considered related to treatment. No other AEs were reported by more than 1 participant in any group. One participant administered placebo experienced a serious AE (grade 3 exacerbation of asthma), which was considered not related to study treatment.

No clinically significant findings were observed for the clinical laboratory tests, vital signs, electrocardiograms, physical and neurological examinations, Columbia Suicide Severity Rating Scale, or Montreal Cognitive Assessment in either the HVs or PD participants. Changes from baseline for the MDS‐UPDRS, Montreal Cognitive Assessment, and Scale for Outcomes in Parkinson's Disease for Autonomic Symptoms did not indicate obvious worsening of PD signs or symptoms in participants who received BIIB054. At day 4, the Nonmotor Symptoms Scale score in the 45‐mg/kg group appeared to be higher than at baseline, but was similar to baseline at subsequent timepoints (data not shown).

### Pharmacokinetics

Overall, the pharmacokinetics of BIIB054 at dose levels of 1 to 135 mg/kg was similar to other immunoglobulin G monoclonal antibody drugs that have minimal target‐mediated CL.^14^ In the HVs and PD participants, serum BIIB054 concentration‐time profiles exhibited a multiphasic decline (Supplementary Fig. 1A,B). In the HVs, maximum serum BIIB054 concentrations were achieved at a median time to C_max_ of 2.48 to 4.27 hours after the start of infusion, and in the PD participants, the median time to C_max_ was 2.07 to 2.54 hours (Supplementary Table 1).

In the HVs, BIIB054 exposure in serum (AUC_0‐inf_, AUC_0‐tlast_) and C_max_ increased in a dose‐proportional manner over doses of 1 to 135 mg/kg (Supplementary Table 1). The terminal elimination phase of the concentration‐time profiles was similar for all doses between 1 to 135 mg/kg, with t_½_ of 27.7 to 34.8 days. The mean CL values were 0.00401 to 0.00542 L/h. The volume of distribution at steady state ranged from 4.34 to 5.25 L, indicating that the distribution of BIIB054 is mostly limited to the vascular space and limited extracellular fluid volume. Interparticipant variability for exposure and pharmacokinetic parameters was low to moderate (Supplementary Table 1). BIIB054 was detectable in the CSF in all cohorts except for the 1‐mg/kg group, where CSF concentrations remained below the lower limit of quantification for all samples (Supplementary Fig. 1C). CSF concentrations generally increased with an increase in dose. The highest CSF concentrations were observed at the last CSF timepoint during week 3 postinfusion.

In the PD participants, serum exposure was nearly dose proportional. Both AUC_0‐inf_ and AUC_0‐tlast_ were 3.2‐fold higher and C_max_ was 2.8‐fold higher at 45 mg/kg versus 15 mg/kg. Other pharmacokinetic parameters were similar for both doses and comparable to the pharmacokinetic parameters for HVs (Supplementary Table 1).

BIIB054 was detectable in the CSF of PD participants at 1 and 4 weeks postdose and increased with dose (Supplementary Fig. 1D). CSF concentrations decreased slightly over time from 1 to 4 weeks postdose.

In the HVs, the average BIIB054 CSF‐to‐serum ratio was approximately 0.2% during week 3 postinfusion, ranging from 0.128% to 0.250%. The CSF‐to‐serum ratio was generally similar across doses (Supplementary Table 2). Observed CSF‐to‐serum ratios in the PD participants were slightly higher than in the HVs. At 4 weeks postinfusion, the mean CSF‐to‐serum ratios were in the range of 0.273% to 0.559% (Supplementary Table 3).

### Immunogenicity

Binding anti‐BIIB054 antibodies were detected predose in 2 HVs who received placebo; 1 remained positive throughout, and the other was negative on day 84. None of the remaining HVs administered BIIB054 or placebo and no PD participants were found to have binding anti‐BIIB054 antibodies at any timepoint up to the end of the study.

### Pharmacodynamics

In the HVs, the plasma α‐syn concentration increased in a dose‐related manner (*P* = 0.0050), and the 135‐ and 90‐mg/kg BIIB054 treatment groups were statistically different from placebo (*P* = 0.0062 and 0.0351, respectively). The mean baseline‐corrected AUC for total α‐syn with BIIB054 showed a significant dose response (*P* = 0.0140) and was greater than with placebo at all doses ≥15 mg/kg, but only significantly different from placebo at the 135‐mg dose (*P* = 0.0241; Supplementary Table 4). In the PD participants, the mean ± standard deviation baseline‐corrected AUC of plasma total α‐syn for BIIB054 45 mg/kg was significantly larger versus placebo (−64.8 ± 760 vs −819.6 ± 2786; *P* = 0.03; Supplementary Table 4).

**Figure 3:**
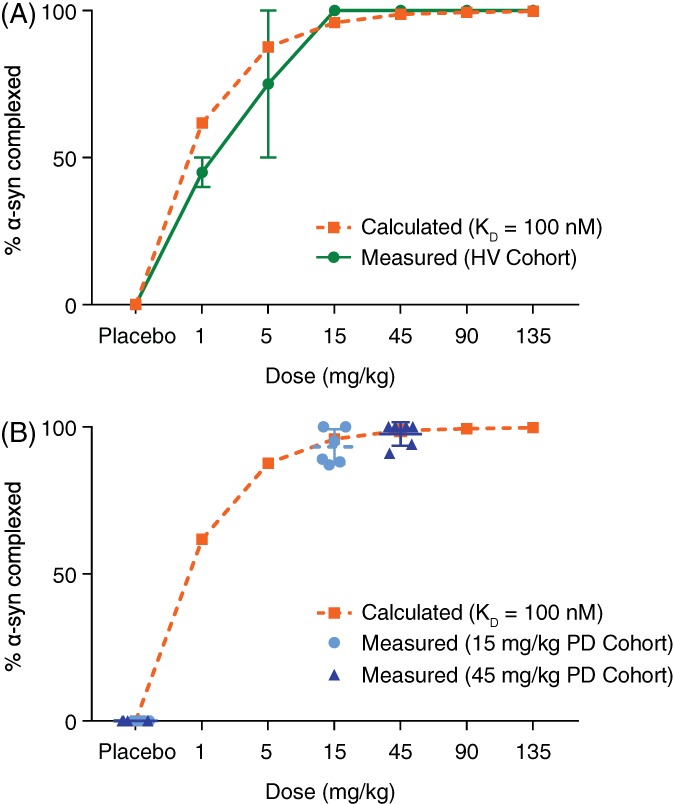
Percent of total α‐syn bound to BIIB054 in plasma at 48 hours postinfusion in (**A**) healthy volunteers and (**B**) participants with Parkinson's disease (PD). α‐syn, α‐synuclein.

Dose‐dependent formation of BIIB054/α‐syn complexes was detected in plasma at 48 hours after infusion. The measured percentage bound showed good correlation with calculated values based on a dissociation constant (K_D_) of 100 nM, the affinity of BIIB054 to monomeric α‐syn (Fig. 3A, Supplementary Materials, and Supplementary Fig. 2).^9^ In the HVs, the percentage of α‐syn bound to BIIB054 reached 100% at doses ≥15 mg/kg (Supplementary Fig. 3). Similarly, all 15‐ and 45‐mg/kg PD participants showed almost complete saturation of the BIIB054/α‐syn complex formation (Fig. 3B).

The BIIB054/α‐syn complex formation was not assessed in the CSF samples because the BIIB054 CSF concentrations are 250‐fold to 400‐fold lower than in serum, far below the K_D_ for binding of BIIB054 to monomeric α‐syn.^9^ No meaningful changes in CSF total α‐syn were observed in the HV and PD participants despite the appearance of numerical increases in α‐syn concentrations at some timepoints (data not shown).

## Discussion

In this first study of BIIB054 in humans, BIIB054 was generally well tolerated at doses up to 90 mg/kg. Although BIIB054 was well tolerated by most HVs in the 135‐mg/kg group, 1 individual in this group experienced a serious AE and another discontinued BIIB054 infusion because of a hypersensitivity reaction. The incidence of treatment‐emergent AEs was comparable across treatment groups in both parts of the study and did not suggest a relationship to dose.

The relatively high frequency of headaches in both the active and placebo groups and their temporal relationship with lumbar puncture suggest that (at least in some individuals) this AE might be procedure related.^15^ Finally, the absence of BIIB054‐binding antibodies is reassuring, although the single dose and small number of participants in this study preclude definitive conclusions about the immunogenicity of BIIB054. Binding anti‐BIIB054 antibodies were detected at baseline in 2 participants who received placebo. Preexisting antibodies in patients without previous drug exposure are frequently observed for biotherapeutics and may represent an adaptive immune response following the exposure to antigens with structural similarities to the biotherapeutic.^16^, ^17^

The most concerning AE observed was the MRI finding consistent with cerebral ischemia. This adverse event occurred after a dose of 135 mg/kg, approximately 3‐fold higher than the highest dose being tested in the ongoing phase II study (NCT03318523). Although neither the proposed mechanism of action of BIIB054 nor the results of preclinical toxicology studies suggest that stroke or other types of vascular disease are likely to be adverse effects of BIIB054, the possibility of a relationship between BIIB054 exposure and this event could not be excluded at this early stage of development. The overall prevalence of incidental or “silent” brain infarctions has been reported to range from 5% to 60%.^18^ The occurrence of this event at the highest dose of BIIB054 administered, together with the participant's absence of cardiovascular risk factors, needs to be considered when evaluating the potential causal relationship between treatment and this event. In addition, it should be noted that exposure equivalent to the 45‐mg/kg dose level has been set as the highest exposure for the currently ongoing phase II clinical trial of BIIB054, based on the projections for aggregated α‐syn to BIIB054 binding saturation in CSF. Also, based on antibody:antigen complex measurements in plasma in this study, BIIB054 to total α‐syn binding in plasma is saturated at this dose. Overall, the 45‐mg/kg dose provides an adequate safety margin when compared with the 135‐mg/kg dose at which this AE occurred.

The pharmacokinetic profile of BIIB054 suggests dose proportionality and similarity to other immunoglobulin G monoclonal antibody drugs with minimal target‐mediated CL.^14^ The relatively long t_½_ of 28 to 35 days supports monthly dosing regimens currently being tested in a phase II trial of BIIB054 (NCT03318523). Interestingly, CSF exposure seemed to be slightly lower in the HVs than in the PD participants, which might point to either age, gender, or possibly disease‐related changes in blood–brain barrier permeability. The current study was not powered to assess these possibilities.

The baseline‐corrected AUC of α‐syn levels in plasma showed a dose‐dependent increase versus placebo at doses ≥15 mg/kg in the HVs and 45 mg/kg in the PD participants. We speculate that the binding of the antibody to α‐syn within the intravascular compartment, where the K_D_ for monomeric α‐syn is exceeded, prolongs α‐syn t_½_ by decreasing its CL and therefore increasing levels. This is further supported by the observation of a dose‐dependent formation of BIIB054/α‐syn complexes in the HV plasma samples and almost complete saturation of BIIB054/α‐syn complex formation in the PD participants. These observations correlate well with the low affinity of BIIB054 for monomeric α‐syn.

The study has some limitations. First, the relatively small sample size and single dose of BIIB054 provide only preliminary assessment of safety and immunogenicity. An ongoing phase II study of BIIB054, with repeated dosing in >300 participants at dose levels up to approximately 45 mg/kg, will provide further characterization of safety and immunogenicity for an extended time period. Second, the pharmacodynamic effects of BIIB054 could only be measured in the intravascular compartment with the currently applied method and not in CSF. The selectivity of BIIB054 for aggregated α‐syn (K_D_ = 0.12 nM) is driven by avidity caused by low intrinsic affinity for monomeric α‐syn (K_D_ = 100 nM) and fast‐on fast‐off binding kinetics.^9^ Even in plasma where BIIB054 concentrations far exceed the K_D_ for monomeric α‐syn, the column‐based method was needed to overcome BIIB054/α‐syn dissociation to accurately quantify complex formation. The 250‐fold to 400‐fold lower BIIB054 concentrations in CSF are well below the K_D_ for monomeric α‐syn and, consequently, complex formation or mobilization of monomeric α‐syn could not be measured with our assays. With an 800‐fold higher affinity for aggregated forms of α‐syn than monomeric forms,^9^ we anticipate that BIIB054 concentrations in CSF will far exceed the K_D_ at doses ≥15 mg/kg and fully engage binding to pathological aggregated forms of α‐syn. However, because aggregated α‐syn concentrations in CSF are much lower than monomeric α‐syn, they currently cannot be measured. We hope that in the near future, the successful development of validated assays for aggregated α‐syn will overcome this limitation and allow the precise estimation of BIIB54/α‐syn complexes and α‐syn aggregates in CSF. Protein‐misfolding cyclic amplification and real‐time quaking‐induced conversion are ultrasensitive protein amplification assays adopted from the prion field that might help demonstrate both α‐syn aggregate engagement and reduction in the levels of free α‐syn by BIIB054 in CSF.^19^, ^20^, ^21^ Finally, given the safety profile of BIIB054 in PD participants at a relatively early disease stage and no complications, it is reasonable to expect a similar safety profile in an advanced PD population, but this remains to be proven.

In summary, this first‐in‐human study of single doses of BIIB054 has demonstrated that it was generally well tolerated and showed an acceptable safety profile at doses ≤90 mg/kg and a dose‐proportional pharmacokinetic profile in both the HVs and PD participants. Evidence of biologic activity of BIIB054 in plasma, with near‐maximal BIIB054 complex formation with α‐syn at both doses tested in a PD population, supports the selection of doses currently being studied in the ongoing phase II study (NCT03318523). Future advances in analytical or nuclear imaging methods may allow more direct measurements of the pharmacodynamic activity of BIIB054 in the central nervous system compartment.

## Author Roles

1) Research project: A. Conception, B. Organization, C. Execution; 2) Statistical Analysis: A. Design, B. Execution, C. Review and Critique; 3) Manuscript: A. Writing of the first draft, B. Review and Critique.

M.B.: 1A, 1B, 1C, 2C, 3A, 3B

L.F.: 1A, 1B, 2C, 3B

S.H.: 1A, 1B, 1C, 2C, 3A, 3B

A.E.: 1C, 3B

N.P.: 1A, 1B, 2A, 2B, 2C, 3A, 3B

M.Y.: 1A, 1B, 2A, 2B, 2C, 3B

M.W.: 1A, 1B, 3A, 3B

E.K.: 1A, 1B, 3A, 3B

E.D.: 1C, 3B

T.F.: 1A, 1B, 1C, 2C, 3B

S.M.: 1A, 1B, 3A, 3B

J.A.: 1C, 3B

I.G.: 1C, 3B

B.P.: 1A, 3A, 3B

Y.L.: 1A, 1B, 1C, 3A, 3B

D.G.: 1A, 1B, 3A, 3B

A.W.: 1A, 3B

J.M.C.: 1A, 1B, 2C, 3B

## Financial Disclosures of all authors (for the preceding 12 months)

M.B. is an employee of and holds stock/stock options in Biogen and is an adjunct associate professor of neurology at New York University School of Medicine. L.F. is a stockholder and employee of Biogen. S.H. is a stockholder and former employee of Biogen. A.E. has consulted for Adamas, Allergan, Amneal, Ipsen, Lundbeck, and Neuroderm and received speaker honoraria from Adamas, Allergan, Arbor, Ipsen, Teva, and US World Meds. N.P. is an employee of and holds stock/stock options in Biogen. M.Y. is an employee of and holds stock/stock options in Biogen. M.W. is a stockholder and former employee of Biogen. E.K. is an employee of and holds stock/stock options in Biogen. E.D. is a stockholder and employee of Biogen. T.F. is an employee of and holds stock/stock options in Biogen. S.M. is an employee of and holds stock/stock options in Biogen. J.A. receives research support from Biogen, AbbVie, Acadia, Neuroderm, Boston Scientific, Impax, and the National Institute of Neurological Disorders and Stroke and received honorarium for consulting or education from Teva, Allergan, Abbvie, and Adamas. I.G. is an employee of Bioclinica. B.P. is an employee of and holds stock/stock options in Biogen. Y.L. is an employee of and holds stock/stock options in Biogen. D.G. is an employee of and holds stock/stock options in Biogen. A.W. is an employee of and holds stock/stock options in Biogen and a stockholder and former employee of Neurimmune AG. J.M.C. is an employee of and holds stock/stock options in Biogen.

## Supporting information


**Appendix S1:** Supporting Information
