# Supplementary material for: Randomized phase I clinical trial of anti–α‐synuclein antibody BIIB054
Source: Mov Disord. 2019 Jun 17;34(8):1154–63. doi: 10.1002/mds.27738 (PMC6771554; doi:10.1002/mds.27738)

SUPPLEMENTARY MATERIALS

FULL INCLUSION AND EXCLUSION CRITERIA

Healthy Volunteers (Cohorts 1 Through 6)

Inclusion Criteria

To be eligible to participate in this study, candidates must have met the following eligibility criteria prior to day 1 or at the timepoint specified in the individual eligibility criterion listed**:**

1. Ability to understand the purpose and risks of the study and provide signed and dated informed consent and authorization to use confidential health information in accordance with national and local subject privacy regulations. If required by local law, candidates must have also authorized the release and use of protected health information (PHI).
2. Healthy adults aged 40 to 65 years, inclusive, at the time of informed consent.
3. All women of childbearing potential and all men were to practice effective contraception during the study and for 6 months after their last dose of study treatment.
4. must have had a body mass index (BMI) from 19 to 30 kg/m^2, inclusive**.**
5. Must have been in good health as determined by the Investigator, based on medical history, physical examination, and 12-lead electrocardiogram (ECG).

Exclusion Criteria

Candidates were excluded from study entry if any of the following exclusion criteria existed prior to day 1 or at the timepoint specified in the individual criterion listed:

1. History of cardiovascular disease (eg, hypertension, arrhythmia, heart failure, long QT syndrome, or other conditions/diseases causing prolongation of the QT/QT interval corrected with Fridericia's formula [QTcF]).
2. Clinically significant (as determined by the Investigator) 12-lead ECG abnormalities, including prolongation of corrected QT interval (eg, repeated demonstration of a QT/QTcF interval >450 millisecond [men] and >470 millisecond [women] before study treatment administration).
3. History of malignancy, including solid tumors and hematologic malignancies (except basal cell and squamous cell carcinomas of the skin that had been completely excised and were considered cured).
4. Clinically significant drug or food allergies, as determined by the Investigator.
5. History of any clinically significant endocrinologic, hematologic, hepatic, gastrointestinal, immunologic, metabolic, urologic, pulmonary, neurologic, dermatologic, psychiatric, renal, or other major diseases, as determined by the Investigator.

6. History of drug or alcohol abuse within the past 5 years, a positive urine drug test, and/or a positive alcohol urine test at the Screening visit or day -1 (as defined by the Investigator).
7. Candidate smoked >5 cigarettes or the equivalent in tobacco daily. Must have been willing to abstain from using tobacco and tobacco-containing products for 72 hours prior to day 1 and during the Inpatient Dosing/Monitoring Period.
8. Treatment with any prescription medication and/or over-the-counter products (excluding acetaminophen, hormone replacement therapy [HRT], and birth control) within 14 days prior to day -1. Routine vitamin therapy was allowed.
9. Surgery within 3 months prior to day -1 (other than minor cosmetic surgery and minor dental surgery, as determined by the Investigator).
10. Clinically significant abnormal laboratory test values, as determined by the Investigator, or any values for alanine aminotransferase (ALT), aspartate aminotransferase (AST), bilirubin, or creatinine that were above the upper limit of normal, any value of hemoglobin that was <12 g/dL for men or <11 g/dL for women, any values for platelets that were below the lower limit of normal, or any clinically significant out-of-normal-range values for white blood cells (WBC) at the Screening visit or day -1.
11. History or positive test result at the Screening visit for hepatitis C virus antibody or hepatitis B virus (defined as positive for hepatitis B surface antigen or hepatitis B core antibody).
12. Known history or positive result for human immunodeficiency virus.
13. Currently active infection or serious infection (eg, pneumonia or septicemia) within the 2 months before day –1, as determined by the investigator.
14. Female candidates who were pregnant, currently breastfeeding, or attempting to conceive during the study.
15. Blood donation (≥1 unit) within 2 months prior to day -1.
16. Vigorous exercise (as determined by the Investigator) within 48 hours of day –2.
17. History of lumbar surgery for any reason (eg, herniated disc) or other contraindications to having a lumbar puncture (LP), including but not limited to chronic back pain sufficient to interfere with activities of daily living on a regular basis; prominent scoliosis; X-ray, magnetic resonance imaging (MRI), or myelographic evidence of significant lumbar spine abnormalities that might have interfered with performance of an LP; history of chronic tension or migraine headaches; or a refractory or prolonged headache or other complication after a prior LP, including that performed at the Screening visit that did not resolve with conservative treatment. Note: all clinically significant (in the opinion of the Investigator) post-LP symptoms must have been resolved before dosing on day 1.

18. Any live or attenuated immunization/vaccination within 2 months prior to first dose or plans for any immunization/vaccination during the study period.
19. Current enrollment or a plan to enroll in any interventional clinical study in which an investigational treatment or approved therapy for investigational use was to be administered within 30 days (or five half-lives of the agent, whichever was longer) before the screening visit and/or participation in any other clinical study involving experimental medications for Parkinson's disease (PD) within 60 days (or five half-lives, whichever was longer) prior to first dose.
20. Inability to comply with study requirements, including the presence of any condition (physical, mental, or social) that prevented the candidate from participating in visits as scheduled.
21. Other unspecified reasons that, in the opinion of the Investigator or sponsor, made the candidate unsuitable for enrollment.

Participants With Early PD (Cohort 7 Only)

Inclusion Criteria

To be eligible to participate in this study, candidates must have met the following eligibility criteria before day 1 or at the timepoint specified in the individual eligibility criterion listed:

1. Ability to understand the purpose and risks of the study and provide signed and dated informed consent and authorization to use confidential health information in accordance with national and local subject privacy regulations. If required by local law, candidates were to also authorize the release and use of protected health information.
2. Adults aged 40 to 80 years, inclusive, at the time of informed consent.
3. All women of childbearing potential and all men were to practice effective contraception during the study and for 6 months after their last dose of study treatment.
4. Must have had a BMI from 19 to 32 kg/m^2, inclusive.
5. Must have met all of the following criteria:
    a. Diagnosis of idiopathic PD (based on the United Kingdom Brain Bank criteria), within 5 years of diagnosis, without motor fluctuations or dyskinesias.
    b. Diagnosis was to be confirmed by the presence of bradykinesia plus one of the other cardinal signs (resting tremor, rigidity) being present, without any known or suspected cause of parkinsonism other than idiopathic PD. The clinical signs were to be asymmetric.
    c. A Hoehn and Yahr stage of ≤2.5 on the modified Hoehn and Yahr scale.

d. Candidates may have been treatment naive and, in the opinion of the Investigator, not expected to require additional symptomatic therapy during the course of the study (ie, no symptomatic treatment for PD was to be initiated during the course of the study). Alternatively, candidates were to continue the following symptomatic therapies if the candidate had been on a stable dose for a minimum of 8 weeks before Screening, and there was no anticipated need for dose adjustments, additional symptomatic therapy, or discontinuation of treatment throughout the duration of the study: up to 5 mg selegiline twice daily, up to 1 mg rasagiline once daily, or up to 25/100 mg immediate-release carbidopa/levodopa three times daily.
e. Except for a clinical diagnosis of PD, the candidate must have been in good health as determined by the Investigator, based on medical history, physical examination, and 12-lead ECG.
f. Up to, but no more than two candidates with scans without evidence of dopaminergic deficit using single-photon emission computed tomography with ioflupane I123 (a dopamine-transporter binding agent marketed as DaTscan™ by GE Healthcare) were to be enrolled in the study.

Exclusion Criteria

Candidates were excluded from study entry if any of the following exclusion criteria existed before day 1 or at the timepoint specified in the individual criterion listed:

Disease Related

1. Presence of drug-induced parkinsonism (eg, metoclopramide and flunarizine), metabolic identified neurogenetic disorders (eg, Wilson's disease), encephalitis, or Parkinson-plus syndromes, or other forms of atypical Parkinsonian syndromes (eg, progressive supranuclear palsy and multiple system atrophy).
2. Presence of freezing of gait.
3. History of stereotaxic brain surgery for PD (eg, pallidotomy, deep brain stimulation, or fetal tissue transplant).

Study Procedures

4. History of lumbar surgery for any reason (eg, herniated disc) that in the opinion of the Investigator would have interfered with or posed risks to the LP procedure or other contraindications to having an LP (low platelet count, anticoagulant therapy [including treatment with aspirin at doses >81 mg per day], and antiaggregants [may have needed 7- to 14-day wash-out]), including but not limited to chronic back pain sufficient to interfere with

activities of daily living on a regular basis; prominent scoliosis; X-ray, MRI, or myelographic evidence of significant lumbar spine abnormalities that might have interfered with performance of an LP; history of chronic tension or migraine headaches; or a refractory or prolonged headache or other complication after a prior LP, including that performed at the Screening visit that did not resolve with conservative treatment. Note: all clinically significant (in the opinion of the Investigator) post-LP symptoms must have resolved before dosing on day 1.

5. Contraindication for MRI (eg, pacemaker, ferromagnetic objects, claustrophobia) or dopamine-transporter imaging (known hypersensitivity to the active substance, any of the excipients, or iodine).

Neurological

6. History within the past 6 months before Screening or evidence of clinically significant (in the opinion of the Investigator) psychiatric illness, including uncontrolled major depression, schizophrenia, bipolar affective disorder, psychosis, or suicidal ideation.
7. Transient ischemic attack or stroke or any unexplained loss of consciousness within 1 year prior to the Screening visit.
8. Brain MRI at Screening showing evidence of acute or subacute micro- or macrohemorrhage.
9. Significant cognitive impairment or clinical dementia that, in the opinion of the Investigator, would have interfered with participation in the study.
10. Clinically significant structural brain disease that would have interfered with study evaluations, as determined by the Investigator.

Cardiovascular

11. History of unstable angina, myocardial infarction, chronic heart failure (New York Heart Association class III or IV), or clinically significant conduction abnormalities (eg, unstable atrial fibrillation) within 1 year prior to Screening.
12. Chronic uncontrolled hypertension (average of three systolic blood pressure/diastolic blood pressure readings at Screening >165/100 mmHg), or any documented systolic blood pressure/diastolic blood pressure reading >180/100 mmHg within 3 months prior to day 1.
13. History of long QT syndrome or other conditions/diseases causing prolongation of the QT/QTcF interval), or clinically significant (as determined by the Investigator) 12-lead ECG abnormalities, including prolongation of corrected QT interval (eg, repeated demonstration of a QT/QTcF interval >450 millisecond [men] or >470 millisecond [women] before study treatment administration).

Hepatic

14. Clinically significant abnormal laboratory test values, as determined by the Investigator, or any values for alanine aminotransferase, aspartate aminotransferase, bilirubin, or creatinine that were above the upper limit of normal, any value of hemoglobin that was <12 g/dL for men or <11 g/dL for women, any values for platelets that were below the lower limit of normal, or any clinically significant out-of-normal-range values for white blood cells at the Screening visit or day –1. Candidates with previously established Gilbert's syndrome and elevated levels of bilirubin consistent with such syndrome were allowed in the study.

Renal

15. Estimated glomerular filtration rate (calculated according to the Modification of Diet in Renal Disease Study equation) <60 mL/min at Screening.

Metabolic

16. Poorly controlled diabetes mellitus, defined as having dosage adjustment of diabetic oral medication within 3 months prior to study treatment administration (day 1) or a glycosylated hemoglobin (HbA_{1c}) value ≥7% at screening.

Oncology

17. History of malignancy, including solid tumors and hematologic malignancies (except basal cell and squamous cell carcinomas of the skin that had been completely excised and were considered cured).

Hypersensitivity

18. Clinically significant drug or food allergies, as determined by the Investigator, including hypersensitivity to radioligands to be used in the study.

Infection

19. History or positive test result at the screening visit for hepatitis C virus antibody or hepatitis B virus (defined as positive for hepatitis B surface antigen or hepatitis B core antibody).
20. Known history or positive result for human immunodeficiency virus.
21. Currently active infection or serious infection (eg, pneumonia or septicemia) within the 2 months prior to day –1, as determined by the Investigator.
22. Any live or attenuated immunization/vaccination within 2 months prior to first dose or plans for any immunization/vaccination during the study period.

General

23. History of drug or alcohol abuse (including the use of marijuana for medicinal purposes) within the past 5 years, a positive urine drug test, and/or a positive alcohol urine test at the Screening visit or day –1 and/or an unwillingness to abstain from these substances during the residential and monitoring periods (as defined by the Investigator).

24. Candidate smoked >5 cigarettes or the equivalent in tobacco daily. Must have been willing to abstain from using tobacco and tobacco-containing products for 72 hours before day 1 and during the Inpatient Dosing/Monitoring Period.
25. Surgery within 3 months prior to day –1 (other than minor cosmetic surgery and minor dental surgery, as determined by the Investigator).

Prior and Concomitant Medications

26. Prior treatment with any biological agent or immunosuppressive medication (including systemic corticosteroids and parenteral immunoglobulins) within 30 days or five half-lives of the agent, whichever was longer, before the Screening visit or during the study.
27. Current enrollment or plan to enroll in any interventional clinical study in which:
    a. An investigational treatment or approved therapy for investigational use was administered within 30 days (or five half-lives of the agent, whichever is longer) before the Screening visit or during the study, or
    b. An experimental treatment for PD was administered within 60 days (or five half-lives of the agent, whichever was longer) before day 1 or during the study.
28. Use of any of the following drugs within 180 days prior to day 1 or during the study: typical or atypical antipsychotics (including, clozapine, olanzapine, flunarizine, and aripiprazole), metoclopramide, or alpha-methyldopa.
29. Use of any of the following drugs within 90 days prior to day 1 or during the study: methylphenidate, cinnarizine, tetrabenazine, reserpine, amphetamine, or monoamine oxidase type A inhibitors (eg, pargyline, phenelzine, and tranylcypromine).
30. Use of any medications for the treatment of comorbid conditions that had not been stable for at least 3 months before day 1 and/or that were not expected to remain stable for the duration of the study.
31. Off-label use of any prescription medications.

32. Candidates who had used over-the-counter medication, including megadose (intake of 20 to 600 times the recommended daily dose) vitamin therapy, within 7 days prior to day 1, or herbal-containing and/or alternative health preparations and procedures within 2 days prior to day 1, unless agreed as not clinically relevant by the Principal Investigator and Sponsor. Routine vitamin therapy was allowed as determined by the Investigator.

Other

33. Female candidates who were pregnant, currently breastfeeding, or attempting to conceive during the study.
34. Blood donation (≥1 unit) within 2 months prior to Day –1.

35. Vigorous exercise (as determined by the Investigator) within 48 hours of Day –2.
36. Inability to comply with study requirements, including the presence of any condition (physical, mental, or social) that prevented the candidate from participating in visits as scheduled.
37. Other unspecified reasons that, in the opinion of the Investigator or sponsor, made the candidate unsuitable for enrollment.

Quantification of α-Synuclein BIIB054 Complex in Human Plasma.

BIIB054 is highly selective for aggregated forms of α-synuclein (α-syn) with a dissociation constant (K_D) <1 nM, and has a lower estimated affinity (~100 nM) for the monomeric nonaggregated form of the protein.[1] Because monomeric α-syn is the predominant form found in biological fluids, BIIB054's weak monomeric affinity and rapid binding kinetics make it challenging to measure BIIB054/α-syn complex in clinical samples. Sample dilution and wash steps used in conventional assays lead to rapid dissociation and result in underestimated amounts of complex measured in these samples. As an alternative approach, we developed a column-based, size exclusion chromatography (SEC) method that separates α-syn complexed to BIIB054 (molecular mass of 160,000 Daltons) from free unbound α-syn (molecular mass of 14,000 Daltons) by differences in their size. The top panel of Supplementary Fig. 2 is a chromatogram showing fractionation of antibody bound α-syn from free α-syn. The blots in the bottom panels of Supplementary Fig. 2 are representative data of individuals from different cohorts demonstrating that α-syn eluted only as monomer in placebo samples, 50% eluted as a complex with BIIB054 and 50% as free monomeric α-syn in cohort 1 samples (1 mg BIIB054/kg) and all of the α-syn in a cohort 3 sample (15 mg BIIB054/kg) eluted as a complex with BIIB054. To increase throughput for the analysis of HV and PD cohort samples, we used SEC fractionation followed by enzyme-linked immunosorbent assay (ELISA; Biolegend) instead of the immunoprecipitation (IP)/western blotting readout for quantification of free and bound α-syn. For the ELISA analyses, only 100 µL plasma samples were applied to the SEC column instead of the 500 µL that was used for western blot quantitation.

Methods

Free α-syn and α-syn/BIIB054 complex were fractionated by SEC on a Superdex® 200 10/300 SEC column (GE Healthcare, 28-9909-44) in phosphate buffered saline (8.9 mM Na_2HPO_4, 1.5 mM KH_2PO_4, 137 mM NaCl, 2.7 mM KCl, pH 7.4) at a flow rate of 30 mL/h. The column was calibrated with α-syn monomer and α-syn/BIIB054 complex. Because α-syn is naturally unfolded and therefore elongated, it has a larger hydrodynamic radius than a globular protein of the same size, which results in the larger apparent molecular weight (~50,000 Da) estimated by SEC than would be predicted by mass alone. Blinded plasma samples from the phase 1 study were thawed and 0.45 µm filtered immediately before use; 500 µL of each sample was loaded onto the column and 0.5-mL fractions were collected. Peak fractions containing free α-syn and the complex were stored at -80ºC for future analysis. α-syn levels in the SEC fractions were measure by IP followed by quantitative western blot analysis. IP studies were performed using anti–α-syn antibody BD42 (BD Transduction Laboratories, 610787) bound to CaptureSelect™ LC-kappa (mur) resin (Thermo Fisher, 191315005). SEC fractions 4 to 10, which contain free and bound α-syn, were each diluted with 1.5-mL of 50 mM Tris-HCl, pH 8.0, 500 mM NaCl, 1% Triton X-100, 10 mM $MgCl_2$, 5% glycerol, 0.5% sodium deoxycholate, 0.1% sodium dodecyl sulfate (RIPA buffer) and incubated with 10-µL resin-containing 4 µg of BD42 for 1 hour at 4°C with continuous mixing. The resin was collected by centrifugation (600 × g, 10 minutes) and washed three times with 500 µL RIPA buffer. Each step was followed by a 10-minute centrifugation step; 8 µL of reducing sodium dodecyl sulfate polyacrylamide gel electrophoresis (SDS-PAGE) sample buffer was added to each resin pellet, and the samples were heated for 3 minutes at 100°C. Samples, including resin, were loaded onto 26-well 10% to 20% Criterion™ TGX Stain-Free™ Protein Gels (Bio-Rad, 5678115) and subjected to SDS-PAGE. Purified recombinant α-syn was loaded onto each gel at 0.2, 0.4, 1, and 2 ng to serve as standards for quantification. Proteins were transferred to 0.2-µm nitrocellulose membranes (Bio-Rad, 170-4159) by a Trans-Blot® Turbo™ Transfer System (Bio-Rad, 1704150) using the programmed

mixed molecular weight protocol. The membranes were blocked in 25 mM Tris-HCl, pH 7.5, 150 mM NaCl, 0.05% Tween-20 (TBST) containing 2% nonfat dry milk (block buffer) for 1 hour, then incubated with 10 µg/mL BD42 in blocking buffer for 1 hour and then with peroxidase-conjugated goat anti-murine antibody (Jackson ImmunoReseach, 115-035-071, 1:5000 dilution) in blocking buffer for 40 minutes, all at room temperature. The membranes were washed three times with TBST for 5 minutes after each incubation step and then incubated with EMD Millipore™ Immobilon™ Western Chemiluminescent HRP Substrate (WBKLS0500) for 5 minutes. The membranes were exposed to X-ray film for 5 seconds. The X-ray films were imaged with a Gel Doc™ EZ System (Bio-Rad, 1708270) and α-syn levels were quantified by the Image Lab software using "volume tool" calculated from the trend line generated with the recombinant α-syn standards. The percentage of complexed α-syn in samples was calculated by dividing α-syn levels in fractions 4 to 7 (shifted in their elution time due to binding to BIIB054) by the total detectable α-syn in fractions 4 to 10.

Reference

1. Weihofen A, Liu Y, Arndt JW, et al. Development of an aggregate-selective, human-derived α-synuclein antibody BIIB054 that ameliorates disease phenotypes in Parkinson's disease models. *Neurobiol Dis* 2019; 124: 276-288.

Supplementary Table 1. Pharmacokinetics in serum of BIIB054 in healthy volunteers and participants with PD

	Healthy volunteers						Participants with PD	
	1 mg/kg	5 mg/kg	15 mg/kg	45 mg/kg	90 mg/kg	135 mg/kg	15 mg/kg	45 mg/kg
Parameter	(n = 3)	(n = 7)	(n = 6)	(n = 6)	(n = 6)	(n = 5)[a]	(n = 6)	(n = 6)
$AUC_{0\text{-inf}}$, h·µg/mL	15,100 (16.6)	70,800 (23.2)	254,000 (14.5)	702,000 (24.1)	1,280,000 (37.1)	2,290,000 (18.4)[b]	241,000 (28.7)	776,000 (23.4)
$AUC_{0\text{-tlast}}$, h·µg/mL	13,800 (15.3)	65,800 (21.4)	232,000 (12.7)	647,000 (16)	1,190,000 (32)	2,080,000 (15.4)[b]	220,000 (26.1)	702,000 (23.5)
C_{max}, µg/mL	30.8 (11.5)	145 (19.7)	531 (7.91)	1570 (16.1)	2920 (12.9)	4310 (20.6)	504 (13)	1400 (24.4)
T_{max}, h[c]	2.50 (2.32-6.02)	2.57 (2.15-6.02)	2.50 (2.50-4.00)	2.75 (2.13-4.00)	4.27 (2.45-14.00)	2.48 (2.13-3.00)	2.54 (1.68-10.05)	2.07 (1.10-3.10)
$t_{½}$, d[d]	31.7 (1.58)	29.5 (5.01)	31.7 (4.98)	30.6 (9.84)	27.7 (10)	34.8 (6.35)	33.1 (7.05)	33.6 (10.7)
CL, L/h	0.00527 (12.3)	0.00521 (24.8)	0.00456 (11.3)	0.00505 (23.8)	0.00542 (58.2)	0.00401 (27.3)[b]	0.00517 (31.6)	0.00465 (32.9)
V_{ss}, L	5.25 (12.9)	4.94 (18.3)	4.6 (14)	4.84 (7.5)	4.61 (10.1)	4.34 (10.9)[b]	5.37 (32.3)	4.94 (25)

$AUC_{0\text{-inf}}$, area under the concentration-time curve from time 0 extrapolated to infinity; $AUC_{0\text{-tlast}}$, area under the concentration-time curve from time 0 to the time of the last measurable concentration; CL, clearance; C_{max}, maximum observed concentration; PD, Parkinson's disease; T_{max}, time to maximum observed concentration; $t_{½}$, elimination half-life; V_{ss}, volume of distribution at steady state. Data are shown as geometric mean and percent coefficient of variation unless indicated otherwise. [a]One participant was administered 163 mL (32%) of the 508-mL infusion because of an adverse event (hypersensitivity reaction) and therefore was not

included in pharmacokinetic analyses. [b]n=4; one participant terminated the study early; no pharmacokinetic samples were collected after the week 3 visit, therefore only C_{max} and T_{max} for this participant were included. [c]Median and range. [d]Arithmetic mean and SD**.**

Supplementary Table 2. Mean (CV%) CSF-to-serum ratio of BIIB054 in healthy volunteers

	BIIB054 dose groups					
	1 mg/kg (n = 3)	5 mg/kg (n = 7)	15 mg/kg (n = 6)	45 mg/kg (n = 6)	90 mg/kg (n = 6)	135 mg/kg (n = 5)[a]
n	0	1	3	3	3	3
8 hours postinfusion, %	NA	0.021	0.006 (27.2)	0.008 (39.0)	0.011 (4.3)	0.007 (26.9)
n	0	4	3	3	3	2
Day 2, %	NA	0.029 (26.7)	0.058 (77.9)	0.048 (49.0)	0.033 (45.4)	0.029 (15.6)
n	0	7	6	6	6	4
Week 3, %	NA	0.183 (69.4)	0.250 (50.4)	0.221 (32.7)	0.180 (61.3)	0.128 (14.3)

CSF, cerebrospinal fluid; CV%, percent coefficient of variation; NA, not available

[a]One participant was administered 163 mL (32%) of the 508-mL infusion because of an adverse event (hypersensitivity reaction) and therefore was not included in pharmacokinetic analyses.

Supplementary Table 3. Mean (CV%) CSF-to-serum ratio of BIIB054 in participants with early PD

	BIIB054 dose groups	
	15 mg/kg (n = 6)	45 mg/kg (n = 6)
Week 1, %	0.368 (67.1)	0.170 (32.4)
Week 4, %	0.559 (74.7)	0.273 (33.0)

CSF, cerebrospinal fluid; CV%, percent coefficient of variation; PD, Parkinson's disease.

Supplementary Table 4. Baseline-corrected log-transformed $AUC_{o\text{-}tlast}$ (h*ng/L) total α-syn in plasma from time zero to week 16

	n	Mean (SD)
Healthy volunteers		
Placebo	14	–583 (2240)
BIIB054		
1 mg/kg	3	–1670 (3110)
5 mg/kg	7	–655 (1170)
15 mg/kg	6	–346 (1510)
45 mg/kg	6	230 (3150)
90 mg/kg	6	355 (2860)
135 mg/kg	5[a]	1810 (569)[b]
Participants with PD		
Placebo	6	–820 (2790)
BIIB054		
15 mg/kg	6	–2930 (3800)
45 mg/kg	6	–64.8 (760)[c]

α-syn, α-synuclein; $AUC_{0\text{-}tlast}$, area under the concentration-time curve from time 0 to the time of the last measurable concentration; PD, Parkinson's disease. [a]One participant was administered 163 mL (32%) of the 508-mL infusion because of an adverse event (hypersensitivity reaction) and therefore was not included in pharmacokinetic analyses. [b]P = 0.0140 for overall dose response and P = 0.0241 for 135 mg/kg versus placebo Missing data was imputed using last observation carried forward for one participant. Excluding this participant's data from the analysis did not change the conclusion. [c]P = 0.03 versus placebo.

Supplementary FIG. 1. Arithmetic mean (±SE) BIIB054 concentrations in serum of (A) healthy volunteers[a] and (B) participants with Parkinson's disease (PD)[a] and in cerebrospinal fluid of (C) healthy volunteers[b] and (D) participants with PD.[b]

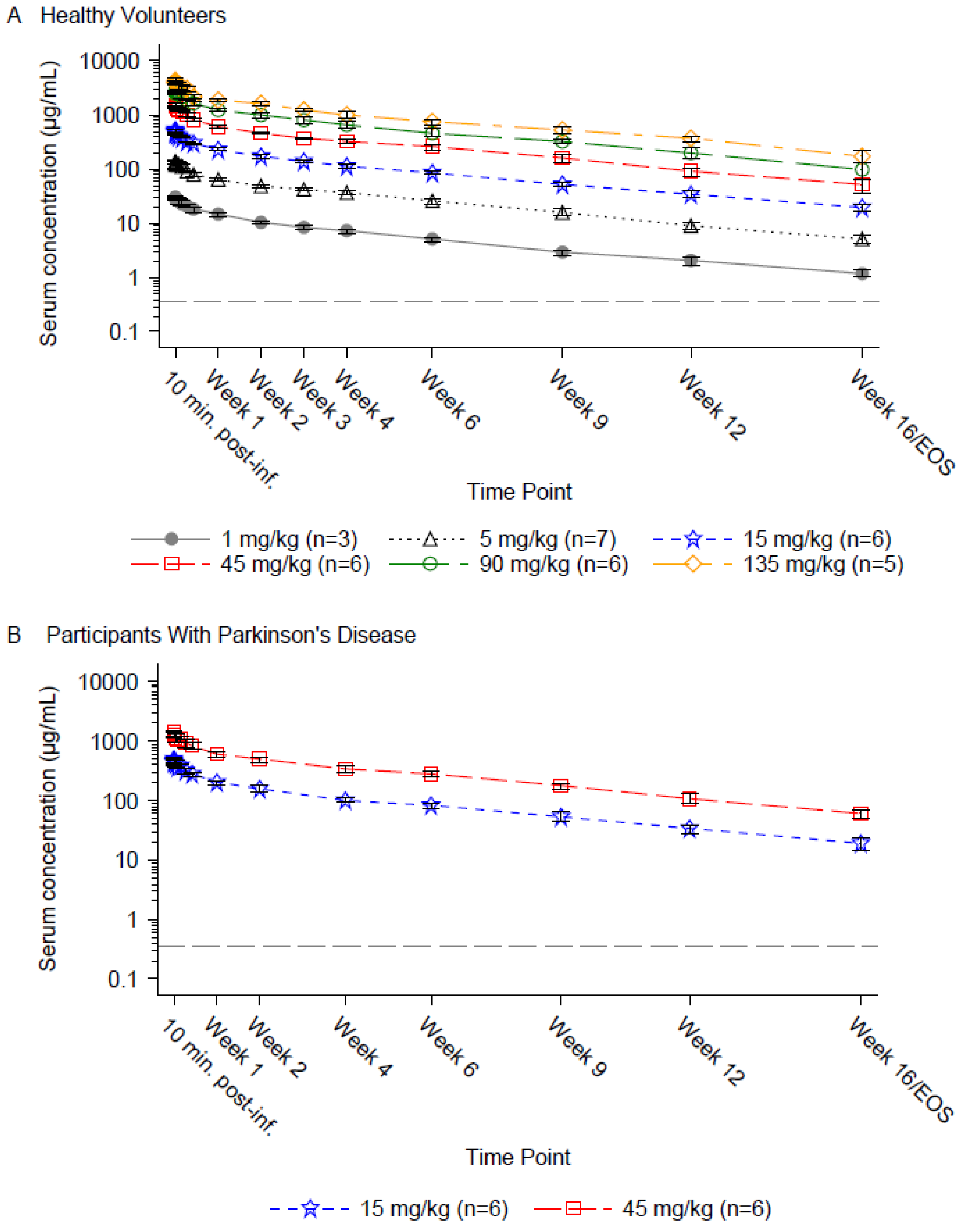

EOS, end of study.
[a]Semilogarithmic scale. Dashed line at 0.375 µg/mL represents lower limit of quantification.
[b]Linear scale. Dashed line at 15 ng/mL represents lower limit of quantification.

C Healthy Volunteers

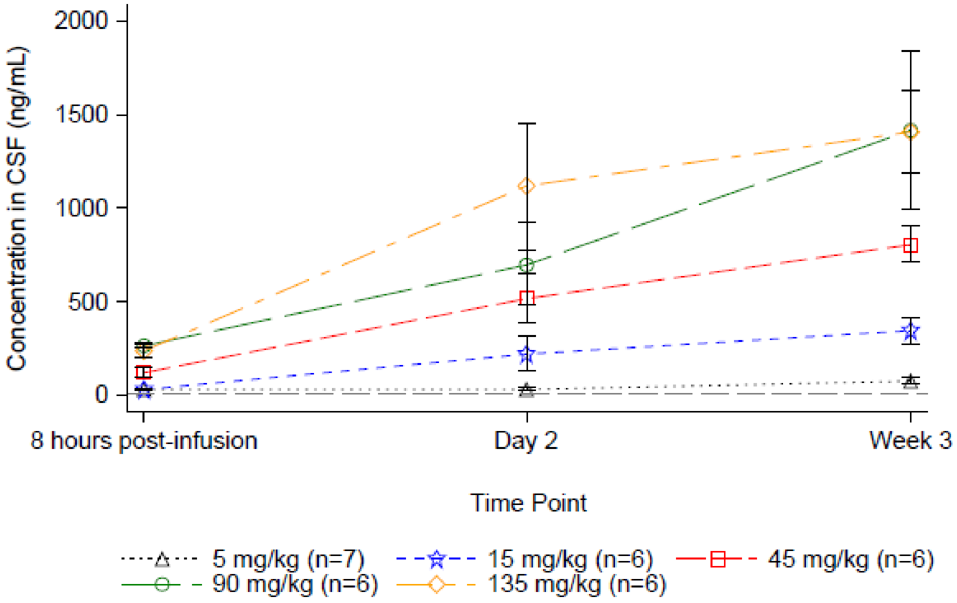

D Participants With Parkinson's Disease

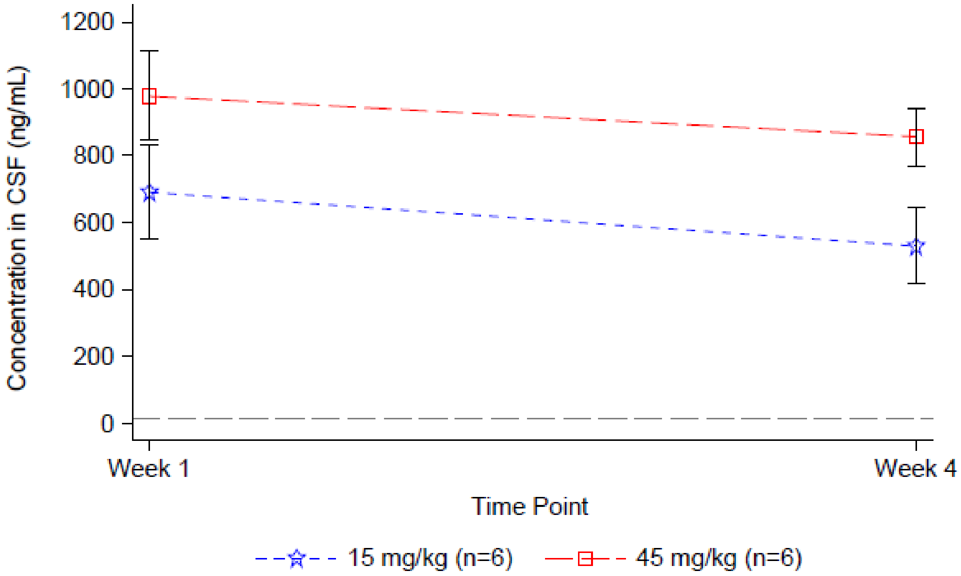

EOS, end of study.

Supplementary FIG. 2 Baseline separation of BIIB054/α-synuclein complex from free monomeric α- synuclein in human plasma samples by size exclusion chromatography (SEC). Blinded plasma samples from the phase 1 study were subjected to SEC and peak fractions analyzed by immunoprecipitation (IP)/western blotting for bound and free α-synuclein. MW, molecular weight.

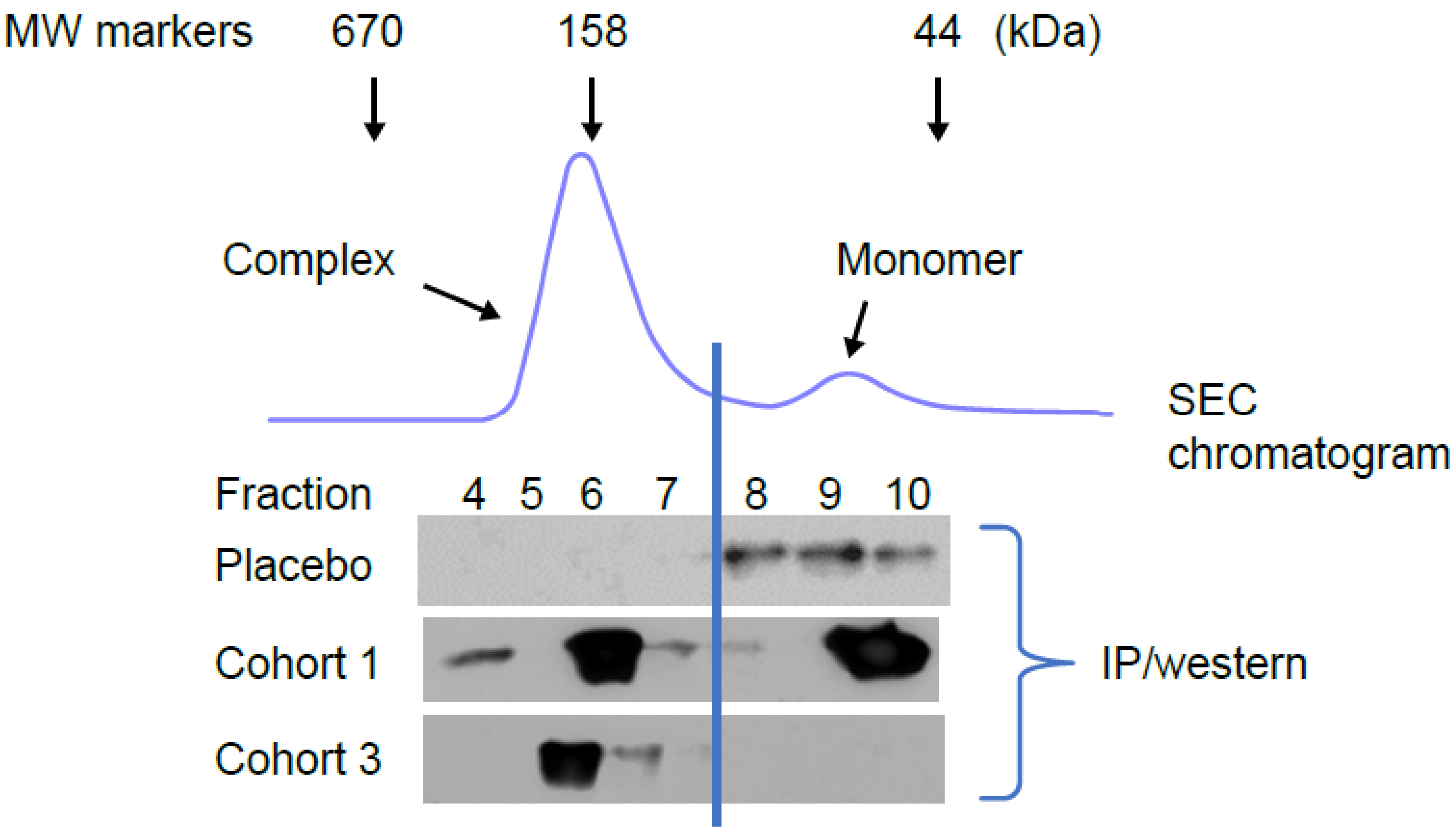

Supplementary FIG. 3. Percent of total α- synuclein (α-syn) bound to BIIB054 in healthy volunteer (HV) plasma at 48 hours postinfusion. Plasma samples were evaluated using the size exclusion chromatography (SEC)-based BIIB054 complex formation assay with ELISA detection described in the supplementary methods except that only 100 μL of plasma was loaded onto the column. Measured values for 44 of the 48 HV plasma samples at each dose are shown in blue diamonds with mean and standard deviation indicated (4 samples were missing due to instrumental failure or insufficient volume for measurement). Predicted values (orange squares) for % α-syn complexed were calculated from measured concentrations of BIIB054 at each dose using the affinity of BIIB054 for monomeric α-syn (K_D=100 nM).[1] The lower than predicted values of complexed α-syn in the 1 and 5 mg/kg cohorts reflect sample dilution during the chromatography resulting from the 100 μL load volume, which did not occur when 500 μL of plasma was loaded (Fig. 3). Sample dilution did not impact the percent of complexes detected in the ≥15 mg/kg cohorts where complex formation was saturated. All 13 placebo samples showed no complex formation, as expected.

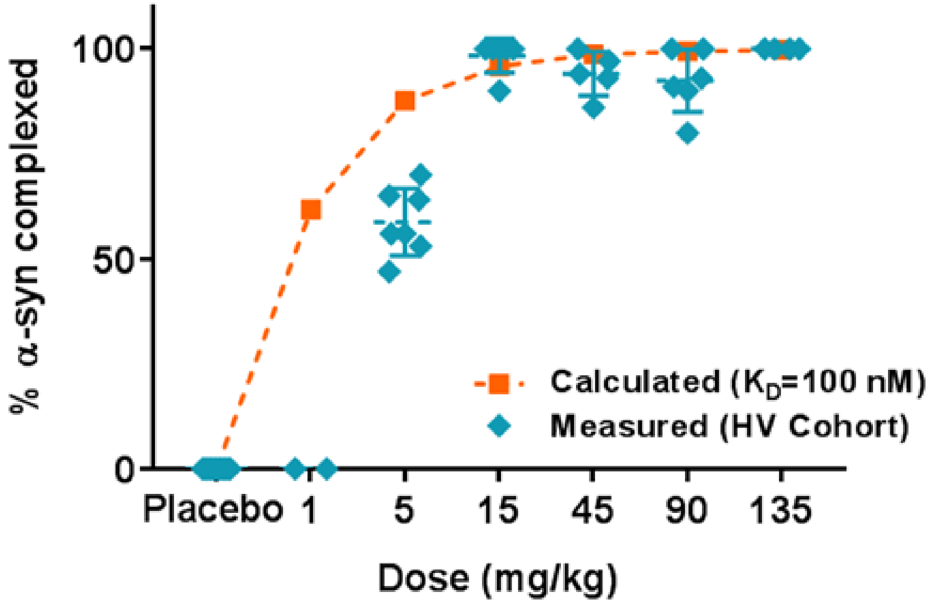

Supplement: Supplementary file 1 — Appendix S1: Supporting Information [file MDS-34-1154-s001.pdf]
